# Angiotensin-(1-7) Receptor Mas in Hemodynamic and Thermoregulatory Dysfunction After High-Level Spinal Cord Injury in Mice: A Pilot Study

**DOI:** 10.3389/fphys.2018.01930

**Published:** 2019-01-11

**Authors:** Anne Järve, Mihail Todiras, Melanie Kny, Falk I. Fischer, Jan F. Kraemer, Niels Wessel, Ralph Plehm, Jens Fielitz, Natalia Alenina, Michael Bader

**Affiliations:** ^1^Max Delbrück Center for Molecular Medicine in the Helmholtz Association, Berlin, Germany; ^2^Partner Site Berlin, German Centre for Cardiovascular Research, Berlin, Germany; ^3^Experimental and Clinical Research Center, Max Delbrück Center for Molecular Medicine, Charité – Universitätsmedizin Berlin, Berlin, Germany; ^4^Department of Physics, Humboldt-Universität zu Berlin, Berlin, Germany; ^5^Partner Site Greifswald, German Centre for Cardiovascular Research, Greifswald, Germany; ^6^Klinik und Poliklinik für Innere Medizin B, Universitätsmedizin Greifswald, Greifswald, Germany; ^7^Charité – Universitätsmedizin Berlin, Berlin, Germany; ^8^Berlin Institute of Health, Berlin, Germany; ^9^Institute of Biology, University of Lübeck, Lübeck, Germany

**Keywords:** blood pressure, heart rate, telemetry, renin-angiotensin system, diurnal rhythm

## Abstract

Spinal cord injury (SCI) above mid-thoracic levels leads to autonomic dysfunction affecting both the cardiovascular system and thermoregulation. The renin-angiotensin system (RAS) which is a potent regulator of blood pressure, including its novel beneficial arm with the receptor Mas could be an interesting target in post-SCI hemodynamics. To test the hypothesis that hemodynamics, activity and diurnal patterns of those are more affected in the *Mas* deficient mice post-SCI we used a mouse model of SCI with complete transection of spinal cord at thoracic level 4 (T4-Tx) and performed telemetric monitoring of blood pressure (BP) and heart rate (HR). Our data revealed that hypothermia deteriorated physiological BP and HR control. Preserving normothermia by keeping mice at 30°C prevented severe hypotension and bradycardia post-SCI. Moreover, it facilitated rapid return of diurnal regulation of BP, HR and activity in wild type (WT) mice. In contrast, although *Mas* deficient mice had comparable reacquisition of diurnal HR rhythm, they showed delayed recovery of diurnal rhythmicity in BP and significantly lower nocturnal activity. Exposing mice with T4-Tx (kept in temperature-controlled cages) to 23°C room temperature for one hour at different time-points post-SCI, demonstrated their inability to maintain core body temperature, *Mas* deficient mice being significantly more impaired than WT littermates. We conclude that *Mas* deficient mice were more resistant to acute hypotension, delayed nocturnal recovery, lower activity and more severely impaired thermoregulation. The ambient temperature had significant effect on hemodynamics and, thus it should be taken into account when assessing cardiovascular parameters post-SCI in mice.

## Introduction

Spinal cord injury (SCI) above the major splanchnic sympathetic outflow impairs cardiovascular homeostasis inducing end organ damage and cardiovascular disease (Garshick et al., 2005; Hou and Rabchevsky, 2014). Cardiac problems contribute to poikilothermia – the inability to respond adequately to thermal challenges and it in turn can cause cardiovascular complications such as bleeding, metabolic acidosis, arrhythmia, decline in cardiac contractility, and exercise-induced hyperthermia (Greene et al., 1989; Colachis, 2002; Price and Campbell, 2003; Tsuei and Kearney, 2004; Price, 2006).

The renin-angiotensin system (RAS), a potent regulator of blood pressure (BP), with its classical harmful components (angiotensin II, AngII and its receptor AT1) becomes activated after high-level SCI (Frankel et al., 1972), initiating target organ damage and possibly autonomic dysregulation (Mathias et al., 1975; Groothuis et al., 2010). However, there are also protective arms of the RAS comprising angiotensin-(1-7), Mas and AT2 receptors counteracting the detrimental effects of the classical RAS, stimulation of which has been proven to be efficient in many cardiovascular disease states (Bader, 2013). Whereas the AT2 receptor has mainly anti-inflammatory and regenerative functions (Steckelings et al., 2010), we have shown recently that Mas plays a role in autonomic dysreflexia following SCI (Jarve et al., 2019). Mas, widely expressed including in the central nervous system (CNS) (Becker et al., 2007), acts protective in the cardiovascular system by lowering BP and suppressing inflammation and oxidative stress. In contrast to AngII, Ang-(1-7), the ligand of Mas, attenuates the nerve stimulation-induced norepinephrine (NE) release (Westfall et al., 2013; Al Dera and Brock, 2014).

Telemetric monitoring of BP in conscious freely moving mice combined with SCI models and transgenic technology would be a potent strategy to evaluate molecular factors involved in cardiovascular dysfunction following SCI. The feasibility of this approach in mice remains to be explored. Therefore, we conducted a pilot study, employing telemetry to characterize acute and chronic hemodynamic reaction to SCI in wild type (WT) and Mas deficient (Mas^-/-^) mice with focus on the development of alterations in diurnal BP and heart rate (HR) rhythms and thermodysregulation. We hypothesized that in the absence of protective Mas the hemodynamic response to SCI would be more severe.

## Materials and Methods

### Animals

Experiments with female WT and *Mas***^-^**^/^**^-^** mice back-crossed into C57Bl/6N background (Walther et al., 1998), weighing 20 to 25 g, and 3–4.5 months of age, with food and water *ad libitum* and a constant 12:12-h light/dark cycle, were conducted according to the National Institutes of Health Guide for the Care and Use of Laboratory Animals, and with the protocols previously approved by the local Animal Care and Use Committee from Berlin LAGeSo (G0132/14).

### Telemetry

We conducted a within animal pre- and post-SCI design, omitting the sham-operated mice in telemetry. All operative interventions were done under intraperitoneal Ketamine (100 mg/kg) – Xylazine (10 mg/kg) anesthesia in combination with Isoflurane (1.5–1.8%) inhalation. As described in detail elsewhere (Gross et al., 2005), the tip of the radiotelemetric device TA11PA-C10 (Data Sciences Int., United States) was implanted into the left femoral artery pointing toward the abdominal aorta. This catheter position is preferred for mice in terms of survival and baroreceptor disturbances compared to a catheter position in the aorta or carotid artery, respectively (unpublished observation) and (Carlson and Wyss, 2000). The transmitter was implanted in a subcutaneous pocket. Recordings were started 7 days after surgery, when hemodynamics had normalized. During the whole study, beat-by-beat arterial BP and HR were continuously monitored 24 h/day. Data were collected using the Dataquest ART system, version 2.1 (Data Sciences International).

### Spinal Cord Injury

On a heating plate (37°C), a dorsal midline incision was made in the superficial muscle overlying a region from the 7th cervical to the 3rd thoracic (C7-T3) vertebrae. The dura was opened at the T2-T3 intervertebral gap and the spinal cord was completely transected using microscissors. Complete transection was confirmed by pulling a needle twice between the rostral and caudal spinal cord ends. Gelfoam was placed above the spinal cord to achieve hemostasis. The muscle and skin were closed with absorbable sutures (Vicryl, 4-0, Ethicon GmbH). Animals received pre-warmed saline (1 ml, s.c.) and recovered on a heating pad for 3–6 h. Body surface temperature was measured by non-contact infrared thermometer held 5 cm from the mouse by pointing the beam at flank. After we had determined that mice do not recover thermoregulation (WT, *n* = 4) the single-housed cages were kept on heating pads (37°C) such that in the cage the temperature was 30°C (*for the telemetry experiment:* WT: *n* = 4, *Mas***^-^**^/^**^-^**: *n* = 4 mice; *for the thermoregulation experiment:* WT: *n* = 10, *Mas***^-^**^/^**^-^**: *n* = 10). For analgesia, mice were treated with Carprofen (4 mg/kg, s.c.) directly after operation and the next day with a 12 h interval or longer if necessary. The bladder was manually emptied three times daily for the entire duration period of the experiment, as bladder function did not recover.

### Assessment of Diurnal Rhythmicity in Hemodynamics and Locomotor Activity

Systolic blood pressure (SBP), heart rate (HR) and gross locomotory activity in the cages were measured and averaged on a 3 h basis (starting 7:30 PM, 10:30 PM; 1:30 AM, 4:30 AM, 7:30 AM, 10:30 AM, 1:30 PM, and 4:30 PM). Hemodynamic variables obtained during the night (7:30 PM–7:30 AM) were compared with values obtained during daytime (7:30 AM–7:30 PM).

### Body-Core Temperature Experiment

In a separate group of mice (WT SCI: *n* = 10, WT sham: *n* = 5, *Mas***^-^**^/^**^-^** SCI: *n* = 10, *Mas***^-^**^/^**^-^** sham: *n* = 5) rectal body temperature was measured by inserting the rectal probe (TMF-3402) connected to the Temperature-Control-Module (TKM-0903, FMI GmbH, Seeheim, Germany) at 9:00 a.m and after 1 or 2 h (as indicated) of group housing at room temperature of 23°C at 4, 10, 17 and 26 days post T4-Tx SCI. Insertion depth was 2 cm and as such reflects core body temperature (Meyer et al., 2017).

### Statistical Analyses

Results are presented as mean ± SEM. Data were analyzed statistically using the GraphPad Prism 5 software (GraphPad Software, CA, United States). One-way and 2-way ANOVA with Bonferroni post-tests were used to detect significant differences in telemetry and thermoregulation as well as body weight loss data, respectively. A value of two-sided *P* < 0.05 was considered statistically significant.

## Results

### Autonomic Regulation at Room Temperature Following SCI

T4-Tx SCI in wild type mice caused systolic blood pressure (SBP) to fall from baseline 110.9 ± 1.3 to 62.4 ± 1.1 mmHg (*p* < 0.0001, Student’s *t*-test) and heart rate (HR) from 607.8 ± 5.4 to 216.2 ± 13.2 bpm (*p* < 0.0001, Student’s *t*-test) within the first 2 (days post-injury) dpi. The surface body temperature of mice measured by a non-contact thermometer fell from 38.2 ± 0.1 to 32.3 ± 1.0°C (*p* < 0.0001, Student’s *t*-test). At 3 dpi we stopped telemetry and kept the mice in the temperature-controlled cage at 30°C for 4 h, after which hemodynamics improved (Supplementary Figure 1). Mice with T4-Tx have thermodysregulation which prevented successful blood pressure assessment unless the mice were kept in temperature-controlled cages.

### Protecting Core Body Temperature Improved Hypotension, Bradycardia and Locomotor Activity Post-SCI

**FIGURE 1:**
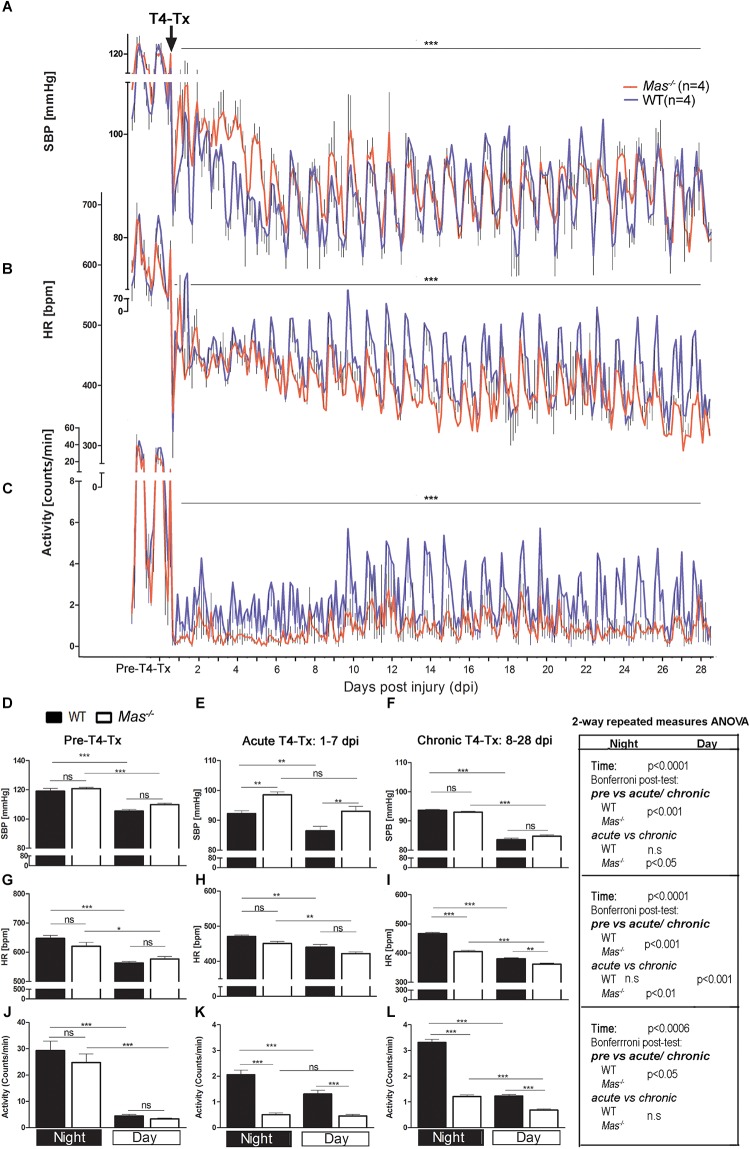
Autonomic functions at 30°C. **(A)** Systolic blood pressure (SBP), **(B)** heart rate (HR) and **(C)** locomotor activity in the cage prior to and after spinal cord injury (SCI). Data are presented as mean values of every 3 h and standard error of the mean (SEM). Significances pre-injury vs. post-T4-Tx for SBP, HR and activity in WT and *Mas*^-^*^/^*^-^ groups, respectively, Student’s *t*-test ^∗∗∗^*p* < 0.001. Baseline, acute phase and chronic phase SBP (**D–F**, respectively), HR **(G–I)** and activity **(J–L)** for daytime and night time. Significances ^∗^(^∗∗^/^∗∗∗^)*p* < 0.05 (0.01/0.001) one-way ANOVA with *Bonferroni*-adjusted *post hoc* analysis, (above bar graphs); right side box for the effect of time point (pre, acute and chronic phase of SCI) 2-way repeated measures ANOVA with *Bonferroni*-adjusted *post hoc* analysis, ns, not significant.

Wild type as well as *Mas*^-/-^ mice kept in the temperature-controlled cages at 30°C had significant, however, less pronounced reduction in the SBP and HR following T4-Tx compared to mice at 23°C (Figures 1A,B). Normothermia, thus, helped to prevent dramatic hypotension and bradycardia following SCI in mice. Interestingly, the SBP fall in the acute phase (1-7 dpi) was significantly smaller in *Mas*^-/-^ mice, however, for the rest of the 4 weeks it was similar in both strains (Figures 1D–F). Heart rate, in contrast, was similar in the acute phase and became significantly higher in WT mice during the chronic phase (Figures 1G–I). Expectedly low locomotor activity post-SCI was observed which remained significantly reduced in *Mas*^-/-^ compared to WT mice in both the acute as well as the chronic phase of SCI (Figures 1C,J–L).

### Normothermia Facilitated the Return of Diurnal Regulation of Hemodynamics

Typical diurnal rhythmicity in cardiovascular control, as characterized by the significantly lower SBP and HR during day when the mice are usually quiescent, was lost acutely following SCI (Figures 1A,B,D–I). Diurnal regulation of SBP and locomotor activity recovered in WT mice kept at 30°C in the acute phase, and in *Mas*^-/-^ mice only in the chronic phase of SCI (Figures 1E,F,K,L). Diurnal rhythmicity in HR at 30°C was present in both strains already in the acute phase of SCI (Figures 1H,I), however, with greater 24-h amplitude in WT. Taken together, preventing hypothermia facilitated the recovery of nocturnal regulation of SPB, HR and activity, although with significant delay in *Mas*^-/-^ mice.

### Mas Protects Core Body Temperature Following SCI

**FIGURE 2:**
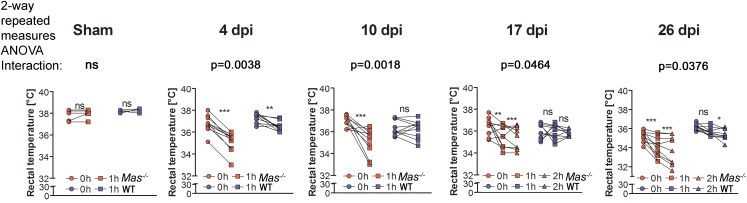
Body core temperature of mice with sham-OP or T4-Tx at indicated time-points post surgery measured prior to (at 30°C) and 1 h after keeping the mice at room temperature (23°C). On 17 and 26 days post injury (dpi) mice were additionally exposed to a 2nd hour at RT. Sham: WT *n* = 5 and *Mas*^-/-^*n* = 5, T4-Tx: WT *n* = 10 and *Mas*^-/-^ *n* = 10. 2-way repeated measures ANOVA, significance of interaction between genotype (WT, *Mas*^-/-^) and time (0, 1, and 2 h at RT) displayed on figure, *Bonferroni*-adjusted *post hoc* analysis, ^∗^(^∗∗^/^∗∗∗^) *p* < 0.05 (0.01/0.001).

In order to determine when thermoregulation might recover, another cohort of mice with T4-Tx were constantly warmed at 30°C and challenged with the 23°C room temperature (RT) for an hour at 4, 10, 17, and 26 days post injury (dpi) before and after which their rectal core body temperature (CBT) was determined. As expected sham-operated WT and *Mas*^-/-^ mice kept their mean CBT during an hour at RT, whereas mice with T4-Tx showed a decrease in CBT (Figure 2). *Mas*^-/-^ mice showed a significantly stronger decrease in CBT during an hour at RT compared to WT mice (Figure 2). Taken together, the ability to maintain CBT would not recover after SCI in the absence of Mas.

## Discussion

Our study is the first to examine resting hemodynamics as well as thermoregulation before and after SCI in WT and *Mas*-deficient mice. We found that (i) T4-Tx SCI in mice caused persistent hypothermia together with severe hypotension and bradycardia; (ii) normothermia improved hypotension, bradycardia and locomotor activity and furthermore facilitated reappearance of their diurnal regulation; (iii) *Mas*^-/-^ mice had significantly impaired thermoregulation post-SCI.

### Thermodysregulation and Its Effect on Hemodynamics

We report that hypothermia post-SCI is drastically affecting the regulation of cardiovascular parameters in mice at room temperature. In contrast to mice, rats present only an acute decrease in body temperature at 1–2 dpi following T3/T4-Tx (Laird et al., 2006; West et al., 2015). Smaller size of mice predestines them for a higher metabolic rate and because of a higher body surface-to-volume ratio they lose proportionally more heat than rats. Consequently, maintaining normothermia by heating the cages improved hemodynamic reactions to SCI and facilitated locomotor activity. Theoretically, the smaller body surface-to-volume ratio of humans should protect them from thermodysregulation, but there are no such data on SCI patients.

### Diurnal Patterns

Thermoneutrality was necessary for rapid restoration of diurnal rhythm in both hemodynamic parameters and in locomotor activity. In comparison, rats with T3/T4 lesion kept in temperature-uncontrolled cages, while normothermic, had rhythmicity returned as late as 2 weeks post-SCI (West et al., 2015). Diurnal desynchrony is involved in the progression of organ dysfunction and development of cardiac disease (Martino et al., 2007; Douma and Gumz, 2017). Quicker return of diurnal rhythmicity in hemodynamics in our study might therefore help to prevent pathology of the cardiovascular system.

### Mas in Hemodynamics

*Mas*^-^*^/^*^-^ mice had higher SBP in acute phase likely reflecting higher vascular tonus (lack of local vasodilation) with diminished local vasodilatory effect of bradykinin and NO as well as Ang-(1-7) (Xu et al., 2008). In addition, circulating AngII activates AT1 receptors expressed in the circumventricular organs, which also drives vasopressin release, and this likely contributes to recovery of BP following SCI (Capone et al., 2012). This could be potentiated in the absence of Mas. Despite of higher SBP *Mas*^-^*^/^*^-^ mice had very low activity suggesting activity-independent circadian regulation of SBP (Sei et al., 1997).

### Putative Role of Mas in Thermoregulation

Mas seems to be required for thermoregulation post-SCI as *Mas*^-/-^ mice could not keep a constant CBT at RT when compared to WT mice. Accordingly, a previous study by our laboratory with an endotoxemia mouse model showed significantly larger loss of CBT in *Mas*^-/-^ mice (Souza et al., 2014). The responsible mechanism in these models might be different and affect thermal sensing, central regulation and efferent axis with thermogenesis and blood flow regulation. Shivering thermogenesis by muscular contraction or non-shivering thermogenesis originating from brown adipose tissue (BAT) could be both impaired in *Mas*^-/-^ mice. Because of paralyzed hind limbs mice could shiver only with the upper body. *Mas*^-/-^ mice lost more skeletal muscle mass as well as body mass following T4-Tx SCI (Supplementary Figure 2) consistent with the effect of Mas deficiency in other muscular dystrophy models (Acuna et al., 2014). Therefore, they might have shivered less effectively and lost more temperature at RT. Indeed, antagonism of Mas leads to highly deteriorated muscular architecture with diminished muscle strength (Acuna et al., 2014). Treatment with Ang-(1-7) prevented atrophy in disuse skeletal muscles, and this effect was absent in the *Mas*^-/-^ mice (Morales et al., 2016). On the other hand, central Ang-(1-7) was reported to increase BAT thermogenesis along with higher level of phosphorylated hormone sensitive lipase which drives BAT lipolysis, processes which conversely could be affected in the absence of Mas (Evangelista and Bartness, 2015). Also mice lacking Mas exhibited a 55% decrease in blood flow in the BAT (Botelho-Santos et al., 2012). Taken together, it seems that Mas is required for thermoregulation involving thermogenesis.

## Conclusion

High-level SCI in mice leads to hypotension, bradycardia and hypothermia. Mas^-/-^ mice had in acute phase less severe hypotension, delayed recovery of diurnal rhythmicity in BP and significantly lower nocturnal activity and could not keep body core temperature as efficiently as wild type mice, furthermore, they had more reduced leg muscles. The major factor influencing hemodynamics and its rhythmicity post SCI was the ambient temperature. Future studies are warranted to explore the effects also in male mice, to find the mechanisms for the differences in the absence of Mas.

## Author Contributions

AJ performed the experiments, analyzed the data, and wrote the manuscript. MT performed the experiments and interpreted the data. MK and FF performed the experiments. JK, RP, and NW analyzed and interpreted the telemetry data. NA and JF revised the manuscript. MB helped by planning the experiments and manuscript revision.

## Conflict of Interest Statement

The authors declare that the research was conducted in the absence of any commercial or financial relationships that could be construed as a potential conflict of interest.
