# Supplementary material for: Angiotensin-(1-7) Receptor Mas in Hemodynamic and Thermoregulatory Dysfunction After High-Level Spinal Cord Injury in Mice: A Pilot Study
Source: Front Physiol. 2019 Jan 11;9:1930. doi: 10.3389/fphys.2018.01930 (PMC6336833; doi:10.3389/fphys.2018.01930)
Supplement: Supplementary file 1 [file Data_Sheet_1.PDF]

Supplementary Fig. 1

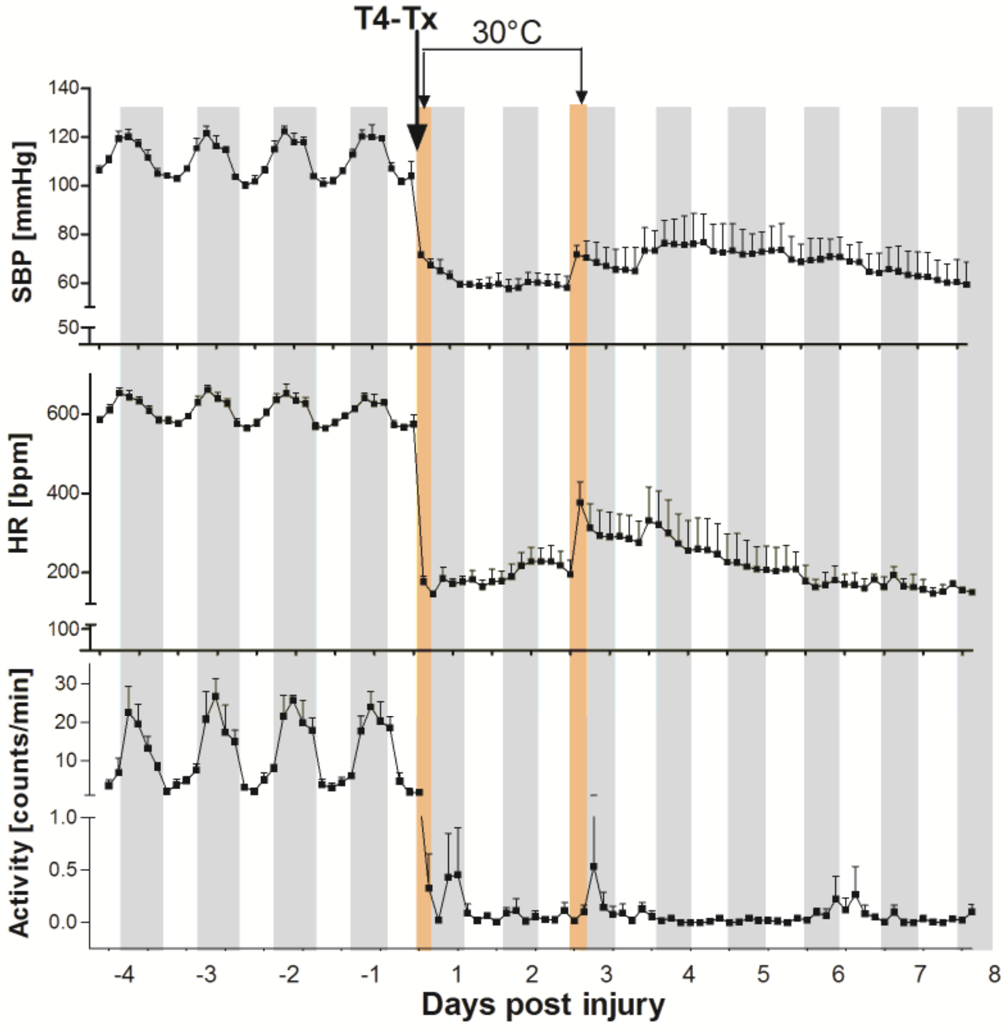

Supplementary Figure 1. A pilot telemetry experiment to monitor systolic blood pressure (SBP), heart rate (HR) and locomotor activity in the cage prior to and after T4-Tx in the WT mice. Time at which the cages were temperature-controlled (temperature in the cages 30°C) is indicated with orange bars and arrows. Mean values of 3 hours are presented. WT n=4. bpm, beats per minute. Night-time is marked with gray bars.

Supplementary Fig 2.

A

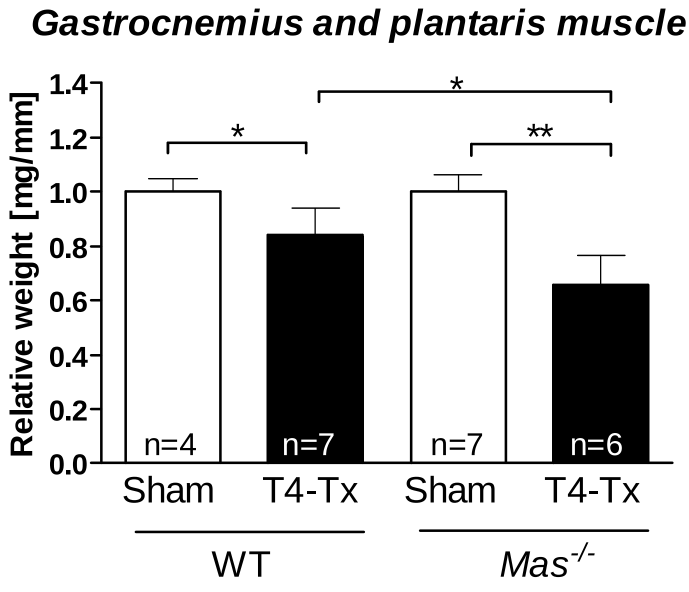

B

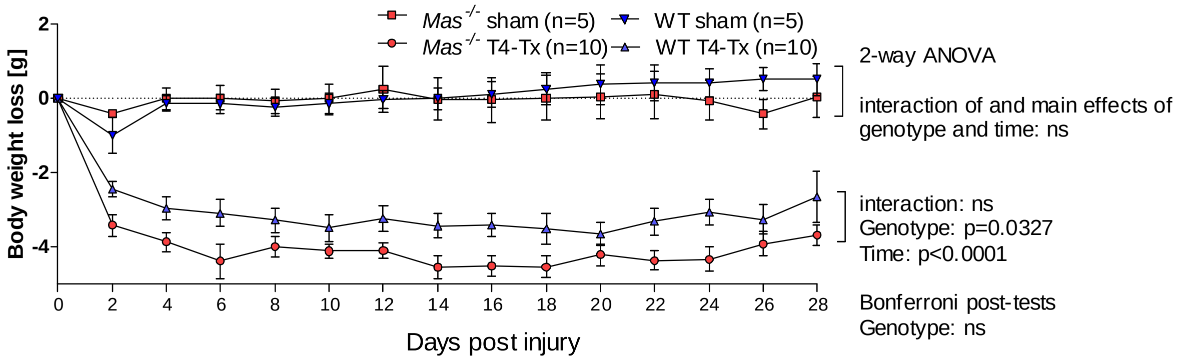

Supplementary Fig 2. (A) Skeletal muscle atrophy after T4-Tx. Weights of Gastrocnemius and plantaris muscle determined at 28 days after surgery in wild type and *Mas*$^{-/-}$ mice. Muscle weight in [g] was normalized to tibia length in [mm]. Data are presented as mean +/- SEM. *p<0.05, **p<0.01. (B) *Mas*$^{-/-}$ mice lost significantly more body weight when compared to wild type mice. ns, not significant.
